# The effect of salt dosing for chytrid mitigation on tadpoles of a threatened frog, *Litoria aurea*

**DOI:** 10.1007/s00360-023-01479-4

**Published:** 2023-02-22

**Authors:** Alex Callen, Ligia Pizzatto, Michelle P. Stockwell, Simon Clulow, John Clulow, Michael J. Mahony

**Affiliations:** 1 Conservation Science Research Group, School of Environmental and Life Sciences, University of Newcastle, Callaghan, NSW 2308 Australia; 2 Centre for Conservation Ecology and Genomics, Institute for Applied Ecology, University of Canberra, Bruce, ACT 2617 Australia

**Keywords:** Batrachochytrium dendrobatidis, Chytrid, Anuran, Tadpole, Salinity, Hormesis

## Abstract

The novel fungal pathogen *Batrachochytrium dendrobatidis* (chytrid) is one of the greatest threats to amphibians worldwide. Small increases in water salinity (up to ca. 4 ppt) have been shown to limit chytrid transmission between frogs, potentially providing a way to create environmental refugia to reduce its impact at a landscape scale. However, the effect of increasing water salinity on tadpoles, a life stage confined to water, is highly variable. Increased water salinity can lead to reduced size and altered growth patterns in some species, with flow-on effects to vital rates such as survival and reproduction. It is thus important to assess potential trade-offs caused by increasing salinity as a tool to mitigate chytrid in susceptible frogs. We conducted laboratory experiments to examine the effects of salinity on the survival and development of tadpoles of a threatened frog (*Litoria aurea*), previously demonstrated as a suitable candidate for trialling landscape manipulations to mitigate chytrid. We exposed tadpoles to salinity ranging from 1 to 6 ppt and measured survival, time to metamorphosis, body mass and locomotor performance of post-metamorphic frogs as a measure of fitness. Survival and time to metamorphosis did not differ between salinity treatments or controls reared in rainwater. Body mass was positively associated with increasing salinity in the first 14 days. Juvenile frogs from three salinity treatments also showed the same or better locomotor performance compared to rainwater controls, confirming that environmental salinity may influence life history traits in the larval stage, potentially as a hormetic response. Our research suggests that salt concentrations in the range previously shown to improve survival of frogs in the presence of chytrid are unlikely to impact larval development of our candidate threatened species. Our study lends support to the idea of manipulating salinity to create environmental refugia from chytrid for at least some salt-tolerant species.

## Introduction

Emerging infectious diseases are one of the greatest threats facing global biodiversity. One potential solution for assisting species impacted by disease in cases where the pathogen cannot be eradicated is to create environmental refuges to help protect declining populations from the impacts of the disease (Puschendorf et al. 2011, 2013). These work by creating environmental conditions that are favourable for the host species but compromise the performance of the disease pathogen, thereby increasing survival for the host (Beadell et al. 2006; Clulow et al. 2018; Stockwell et al. 2012; van Riper et al. 1986; Woodhams et al. 2011). Perhaps one of the best examples of this phenomenon relates to *Batrachochytrium dendrobatidis* (Bd), the amphibian chytrid fungus, a waterborne fungal pathogen that causes the disease chytridiomycosis, responsible for the rapid decline of more than 500 amphibian species globally (Bower et al. 2017; Scheele et al. 2019b). The devastating impacts of chytrid on global amphibian diversity are due to its ability to survive in fresh water and moist soil without a host (Johnson et al. 2003) and its broad host range (Berger et al. 1999). However, chytrid-impacted species have been found to persist or even recover in small pockets which have been linked to certain environmental conditions that are unfavourable to the chytrid pathogen, such as elevated temperature, pH or water salinity (Heard et al. 2015, Stockwell et al 2015, McKnight et al. 2017, Turner et al. 2021). This gives rise to the exciting possibility of mitigating chytrid impacts at a landscape scale by creating or enhancing natural disease refugia through simple habitat manipulation (Clulow et al. 2018; Heard et al. 2018; Scheele et al. 2019a, b; Scheele, Pasmans et al. 2019b; Stockwell et al. 2015).

The salinity tolerance of chytrid in culture is known (Johnson et al. 2003; Stockwell et al. 2012), and habitat manipulations to assess the ability of increased salinity to mitigate chytrid impacts on susceptible frogs has been trialled in laboratory (Stockwell et al. 2015), mesocosm (Clulow et al. 2018) and field scale experiments (Klop-Toker et al. 2021). Salinity levels of 2 ppt significantly reduce the prevalence and infection load in green and golden bell frogs (*Litoria aurea)* compared to those exposed to 0 ppt controls (Stockwell et al. 2012). Pathogen transmission between infected and uninfected individuals of the same species was reduced at 3.5 ppt, improving survival in a wild population raised in outdoor mesocosms (Clulow et al. 2018a). Like many coastal frogs, *L. aurea* occupies ecotonal habitats between salt and freshwater environments and these previous findings suggest that small manipulations of water salinity could be a useful strategy for mitigating chytrid in the landscape of such species. Indeed, the range contraction observed in *L. aurea* and other closely related bell frogs (e.g. *Litoria raniformis*) towards the coastline following the arrival of chytrid strongly suggests that environments with elevated salinity may be a key factor in fighting the disease (Mahony et al. 2013). However, there is debate about the effects of increasing salinity on the vulnerable larval phase for many frog species threatened by chytrid (Christy and Dickman 2002) and the suitability of salinity manipulations to mitigate chytrid at the landscape scale remains untested.

If salt is to be an effective landscape agent against chytrid, its concentration must not be detrimental to the vulnerable larval stage of amphibians. Numerous studies have investigated the response of both fresh water and salt-water adapted larval anurans to differing salt concentrations as static treatments (Alexander et al. 2012; Chinathamby et al. 2006; Christy and Dickman 2002; Kearney et al. 2012). However, few studies have investigated how tadpoles respond to complex salinity fluctuations that occur during seasonal climatic regimens such as pond drying, particularly in species that may exhibit local or genetic adaptation to salinity (Kearney et al. 2012; Wu et al. 2014). Fluctuating salt concentrations likely represent the environmental conditions that tadpoles experience in their natural habitat. Therefore, understanding developmental responses to low and high salinity and their effects on major life history traits is important before proceeding with landscape-scale salinity habitat manipulations. To our knowledge, there is no published study investigating the response of tadpoles of chytrid-susceptible amphibians to a simulated salt dosing regimen that would reduce the prevalence of infection in adult populations.

We aimed to fill this knowledge gap by assessing how tadpoles of a candidate species for salinity-based refugia creation to mitigate chytrid would respond to manipulated salinity levels. We raised tadpoles of the chytrid-susceptible frog *L. aurea* in low (up to 2 ppt) and high (up to 6 ppt) salinity treatments under three salt dosing regimens (constant, increasing and pulse). We compared tadpole survival rates, time to and size at metamorphosis and compared them to tadpoles reared in rainwater. We also assessed post-metamorphic fitness using a jump test to determine whether exposure to elevated salinity in the tadpole stage had flow-on effects across the lifespan.

## Methods

Tadpoles were obtained from a captive breeding colony of *L. aurea* at the University of Newcastle, with founding stock collected from Kooragang Island in the Hunter River estuary on the NSW mid-north coast, Australia. Tadpoles were reared in outdoor mesocosms maintained with rainwater from the time of spawning until the commencement of the experiment at Gosner stage 26 (Gosner 1960). Rainwater was selected as the control environment for the experiment as tadpoles of many amphibians are successfully reared in this medium (Iwai et al. 2012; Pizzatto et al. 2016a, b), including those of *L. aurea,* as part of our long-term captive breeding project.

**Fig. 1:**
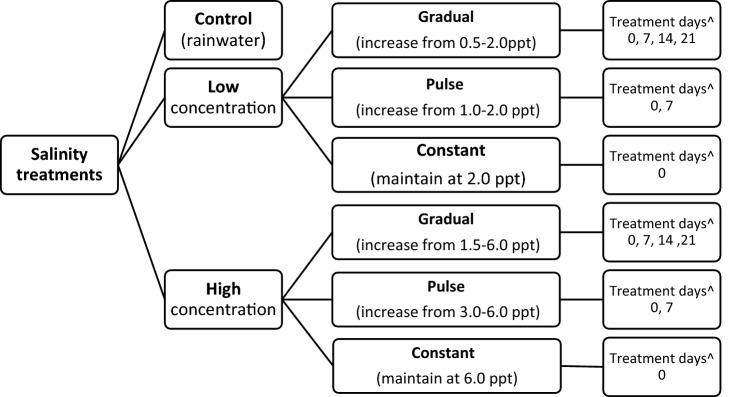
Salinity treatments (control, low and high) in the laboratory experiment with nested gradual, pulse, and constant treatments. ^Treatment days are the days when additional salt was added to each group from the first day (day 0) of the experiment

One hundred and forty tadpoles were randomly selected from three clutches, representing 20 replicates in six treatment groups and a control group. Tadpoles were acclimatised in random groups of 35 tadpoles in 7.5 L plastic tubs filled with rainwater for 48 h prior to the commencement of the experiment. Each tadpole was then individually housed throughout the experiment in a clear plastic container (170 mm × 120 mm × 70 mm) in 600 ml of rainwater maintained to that volume (mean water temperature 22.60 ± 0.86 °C SD). Each container was supplemented with marine-derived sea-salt comprising a minimum of 99.5% sodium chloride, 0.2% moisture and 0.3% insoluble matter (Olsson’s Pacific Dairy Salt, Yennora, NSW) according to the experimental design in Fig. 1. They were kept inside temperature-controlled cabinets (Thermoline Scientific; mean ambient air 24 ± 0.77 °C SD) and exposed to a day/night routine typical of Australian Eastern Daylight Time (AEDT) of 15 h light/9 h dark. Individual housing was used to ensure the independence of individual responses to treatments and to prevent any positive or negative effects associated with intra-specific competition, facilitation or other interactions between individuals during larval development (Kearney et al. 2012). Tadpoles were fed thawed lettuce ad libitum*,* and half the water was changed weekly.

The experimental design consisted of three qualitative salinity treatments: pulse, gradual and constant, and a rainwater control (Fig. 1). The pulse salinity treatment simulated the manual addition of salt to a wetland as previously trialled for chytrid management (Stockwell et al. 2012). The gradual salinity increase and constant salinity treatments reflected the natural environmental conditions under which tadpoles of this species are commonly found (Hamer et al. 2002). The gradual salinity increase treatment simulated wetland evaporation over summer, and concentrations were based on field monitoring of natural wetland levels on the Ash-Kooragang Island system (Hamer et al. 2002). The constant salinity treatment reflected stable environmental conditions rarely reported, but that may occur in permanent wetlands where evaporation effect on total water volume (and hence salinity) is minimal. Each qualitative treatment consisted of a low and high salinity category, resulting in six treatments: pulse low (beginning at 1 ppt and increasing to 2 ppt on day 7) and pulse high (beginning at 3 ppt and increasing to 6 ppt on day 7), gradual low (increasing in 0.5 ppt increments every 7 days from 0.5 ppt to 2 ppt, for 21 days), gradual high (increasing in 1.5 ppt increments every 7 days from 1.5 ppt to 6 ppt, for 21 days), constant low (maintained at 2 ppt throughout the experiment), and constant high (maintained at 6 ppt throughout the experiment) (Fig. 1). All salt additions were completed by 21 days. Control tadpoles were housed under the same conditions and for the same period as treatment groups but remained in rainwater (mean salinity = 0.1 ± 0.07 ppt SD) throughout the study.

Snout-vent length (SVL), total length and body mass were measured for each tadpole prior to being randomly assigned to a treatment group and then weekly until complete metamorphosis (Gosner stage 46) using vernier callipers and digital scales (to the nearest 0.1 mm or mg). The number of tadpoles alive and the number metamorphosed were recorded daily for each group, together with salinity and water temperature using a hand-held metre (EUTECH Testr35). Gosner developmental stage was recorded weekly for each individual (Gosner 1960). The larval component of the experiment lasted 78 days, determined as the time taken for the last tadpole to reach metamorphosis. Final mass and SVL were recorded as each individual reached Gosner stage 46 (complete tail resorption).

Body mass, SVL and right tibia were immediately measured for each individual before locomotor performance testing as juvenile frogs. Locomotor performance testing occurred at an ambient air temperature of 25 °C. It was performed by dipping the ventral surface of the frog in water (so that each jump landing spot would be marked), then placing the frog on a piece of wooden board and prompting the frog to jump by gently touching its vent with the back of a pencil. The distance between each jump for 10 consecutive jumps were measured for each individual using a ruler.

### Data analysis

Tadpole development was analysed as four parameters: (1) survival—the number of tadpoles alive at metamorphosis, (2) time to metamorphosis (number of days from the start of the experiment), (3) mass at metamorphosis, and (4) growth rates, as body mass per week.

Survival was analysed using the Kaplan–Meier estimator of survival. Differences in time to metamorphosis among treatments and the control were assessed using Kruskal–Wallis. Differences in log-transformed mass at metamorphosis among treatments were assessed using analysis of covariance (ANCOVA), with salinity as the independent variable and time to metamorphosis as the covariate. Weekly growth rates were compared for three-time periods (1st, 2nd and 3rd experimental weeks) using ANCOVAs, using initial mass at each of the three-time periods as the covariate. Multiple linear regression was calculated with partial residual plots to examine the relationship between mass and salinity (Larsen and McCleary 1972). Analyses of weekly growth rates were limited to the first three weeks of data collection as many individuals commenced metamorphic climax after that period (Gosner Stage 42), which results in loss of body mass (Wilbur and Collins 1973). The effect of different treatments on post-metamorphic locomotor performance was analysed using ANCOVA to test the mean jump distance of juvenile frogs (based on 10 consecutive jumps). Length of right tibia was a covariate to account for body size effect, after length of right tibia, SVL and body mass were incorporated into a multiple regression to determine the most effective morphometric predictor of locomotor performance. Differences between the control group and treatments were assessed using Dunnett’s test (Ruxton and Beauchamp 2008). Results are presented as means and standard errors, and *p* < 0.05 was set as the statistical significance level in all analyses. All statistical analyses were conducted using JMP 11 (SAS Institute Inc., Cary, NC, 1989–2007).

## Results

### Tadpole survival and development

**Fig. 2:**
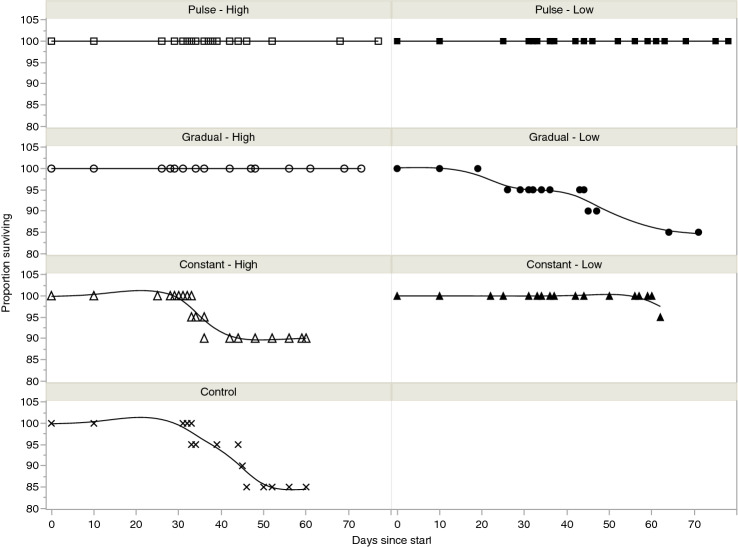
Proportion of *Litoria aurea* tadpoles surviving in each treatment. Line terminations indicate the time at which the last tadpole in each treatment metamorphosed. Tadpole survival ranged from 85–100% and was not significantly different for treatments when compared with the control (*χ*^2^ = 9.72, *df* = 6, *p* = 0.13). Lines represent the survival curve estimated by the probability of occurrence of death at each time point

Tadpole survival ranged from 85 to 100% and was not significantly different for salinity treatments compared to the control (*χ*^2^ = 9.72, *df* = 6, *p* = 0.13). All tadpoles survived salinity increases in the first three weeks of the experiment irrespective of treatment type. Survival in the control group (rainwater) was 85%. Survival in the high salinity treatments was above 90%, while in low salinity treatments, it was above 85% (Fig. 2). Time to metamorphosis also did not differ for treatments compared to the control (*χ*^2^ = 8.86, *df* = 6, *p* = 0.19).

**Fig. 3:**
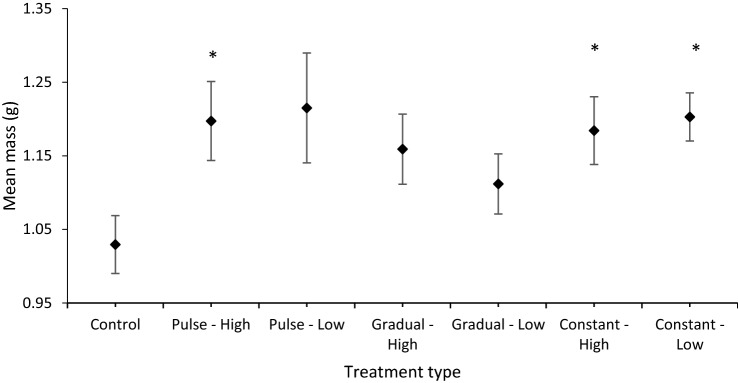
Mean body mass (g) of tadpoles at metamorphosis when raised in high or low salinity, with gradual, pulse or constant salt regimens, compared to the control (rainwater). Diamonds represent means, and whiskers represent standard errors. Treatments significantly different (*p* < 0.05) to the control are labelled with an *

There was a significant difference in body mass of tadpoles at metamorphosis in salinity treatments when compared with the control (ANCOVA *F*_1,6_ = 2.54, *p* = 0.02). Tadpoles exposed to pulse high, constant high and constant-low treatments had significantly greater body masses when compared with the control group (*p* < 0.01 in all cases) (Fig. 3). The final mass in those groups was 15–17% greater than the control group.

Time to metamorphosis was positively related to mass at metamorphosis (ANCOVA *F*_1,6_ = 29.53, *p* < 0.01). There was no interaction between time to metamorphosis and treatment (ANCOVA *F*_1,6_ = 0.74, *p* = 0.62). There was no difference in the tadpole mass of the treatment groups when compared with the control at the start of the experiment (ANOVA *F*_1,6_ = 1.58, *p* = 0.17).

**Fig. 4:**
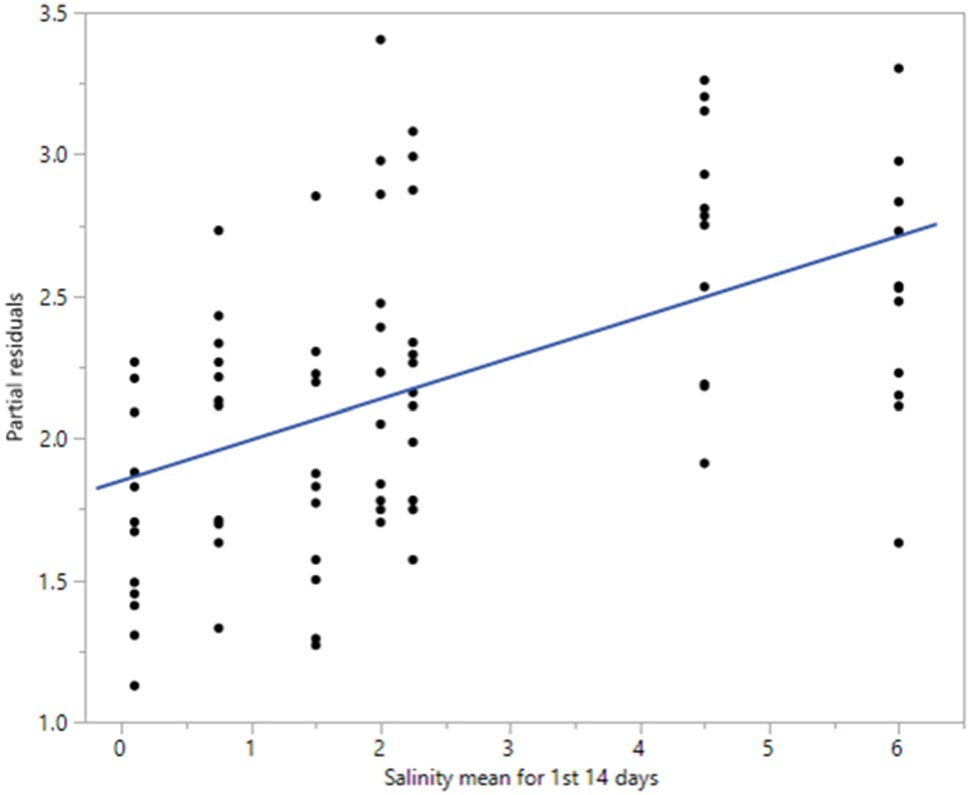
Partial residual plot showing the effect of salinity averaged over the first 14 days of the experiment, from the regression adjusted for the mass at time 0. A constant (1.1) was added to the Y axis to reflect the median mass at time 0. The blue line shows the linear trend fitted to the data

There was a significant difference in mean body mass after the first seven days of the experiment (ANCOVA *F*_6,138_ = 104.12, *p* < 0.01) and between seven and 14 days (ANCOVA *F*_6,72_ = 3.02, *p* = 0.01), but not between days 14 and 21 (ANCOVA *F*_6,65_ = 1.13, *p* = 0.36). There was a significant association between salinity and tadpole mass after 14 days (*F*(2) = 15.43, *p* < 0.0001), suggesting mass at day 0 and mean salinity were significant predictors of mass after 14 days (Fig. 4). This accounted for about 29% variation in body mass in tadpoles (*R*^2^ = 0.286), with the effect holding constant throughout the remaining experimental period, irrespective of changes in salinity treatment regimen (Fig. 4). The regression equation suggested that tadpole mass increases by 0.46 g for each of gram of starting mass and 0.14 g for each one ppt increase in salinity.

### Post-metamorphic locomotor performance

**Fig. 5:**
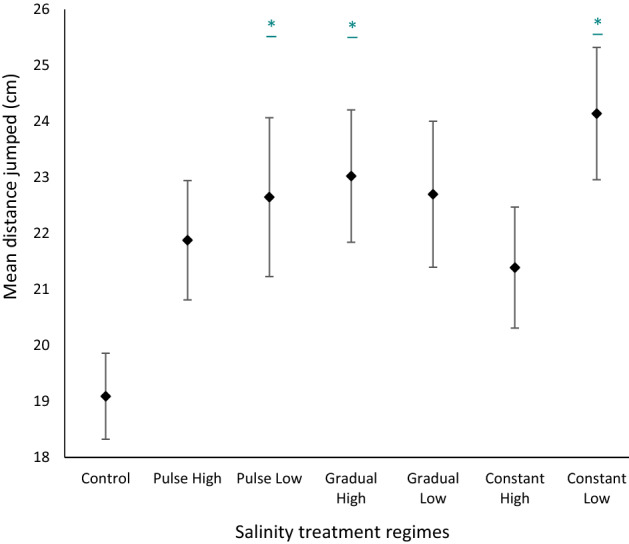
Mean distance jumped by post-metamorphic frogs raised in high or low salinity, with gradual, pulse or constant salt regimens, compared with the control (rainwater). Diamonds represent means and whiskers represent standard errors. Treatments significantly different (*p* < 0.05) to the control are labelled with an *

Right tibia was significantly positively correlated with mean jump distance (ANCOVA *F*_1,6_ = 3.10, *p* = 0.01) and was the only morphometric parameter that was a significant predictor of jump performance (*r*^2^ = 0.36, df = 1, *p* < 0.01). Mean jump distance was significantly different among salinity treatments (ANCOVA *F*_1,6_ = 3.21, *p* = 0.01), with tadpoles exposed to gradual high, pulse low and constant-low treatments jumping greater mean distances than the control group (*p* < 0.01, *p* = 0.01 and *p* = 0.01, respectively) (Fig. 5).

## Discussion

We did not find any negative effects of salt dosing at any concentrations tested on tadpole survival or development, or on the locomotor performance of the post-metamorphic frogs. These findings suggest that water salinity up to 6 ppt may be an effective landscape feature to mitigate the effects of the chytrid pathogen on *L. aurea* without affecting their larval life stage. Tadpoles in all salinity treatments metamorphosed at a similar time to control tadpoles raised in rainwater. The addition of salt did not lead to a decrease in body mass; the addition of salt led to increased body mass in some salt treatments. Finally, locomotor performance in post-metamorphic frogs reared as tadpoles in salinity treatments was similar or better than tadpoles reared in rainwater.

Results from previous studies that examined the effect of salinity on tadpoles in *L. aurea* were also variable, which provided part of the justification for the current study. If salinity is to be used on a landscape scale to mitigate chytridiomycosis in *L. aurea* (Stockwell et al. 2015), we need to be sure it does not negatively impact the tadpole stage. Earlier studies of the influence of salinity on *L. aurea* tadpoles observed slower growth at a constant exposure at 1.87 ppt and above, with loss of body mass when exposure occurred for longer than three weeks (Christy and Dickman 2002). While similar negative growth responses of tadpoles to salinity have been documented in a range of northern hemisphere frogs, mean body masses were comparable with controls (Chinathamby et al. 2006). Variable results suggest that the interaction between larval phenotype and salinity may be complex, dependent on environmental concentration, species, geography, and even populations of the same species (Dananay et al. 2005; Hua et al. 2013; Kearney et al. 2012).

While we found no toxic effect of salinity levels up to 6 ppt for *L. aurea* in our study, there have been mixed results on larval anurans in other studies. Sodium chloride salts used for de-icing roads in the northern hemisphere have reportedly increased the salinity of adjacent roadside ponds to between 2.114 ppt (Brady 2013) and 8.75 ppt (Kaushal et al. 2005), resulting in tadpole mortality, mutation, faster metamorphosis and smaller body size in several native species (Dananay et al. 2005; Gomez-Mestre et al. 2004; Hawley Matlaga et al. n.d.; Kearney et al. 2012). A recent meta-analysis of anuran amphibian tadpole responses to salinity identified an LC50 (lethal concentration invoking a 50% mortality) of approximately 5.5 ppt (Albecker & McCoy 2017). These results highlight that salt sensitivity may be species specific and should be carefully considered before deliberately raising salinity.

While we did not observe any obvious negative toxic effects of salinity levels up to 6 ppt on tadpoles, we did observe some seemingly positive effects in some salt treatments. These included greater tadpole body mass or better locomotor performance post-metamorphosis. One possible explanation for this seemingly paradoxical result could be due to a hormetic response to some salt concentrations (Calabrese & Baldwin 2002; Mattson 2008). Hormesis is a dose–response phenomenon whereby low concentrations of a chemical can stimulate positive physiological responses but higher doses lead to negative effects (Constantini 2014). Hormesis has been observed in larval amphibians in response to nitrate and other contaminants, where low levels stimulate organism growth, but high levels inhibit growth (Chinathamby et al. 2006; Smith et al. 2006; Radovanovic et al. 2021). Hormetic responses by organisms are important for identifying the thresholds at which contaminants induce toxic responses (Constantini 2014). There is growing evidence to suggest that exposure to contaminants at low concentrations that induce stimulatory or beneficial effects may favour environmental priming for the development of a biological shield (Constantini 2014; Agathokleous and Calabrese 2019)—the physiological preparation for stressors later in life to improve survival or fitness. In many organisms, this stimulatory influence results in increased growth. For example, when tadpoles of grey treefrogs (*Hyla versicolor*) were exposed to increasing concentrations of chloride ions, a larger size at metamorphosis was observed (Brand et al. 2010; Van Meter et al. 2011). Similar results were observed in southern brown treefrogs (*Litoria ewingii*) (Chinathamby et al. 2006). Our results show a positive relationship between some levels of salinity and tadpole body mass up to 6 ppt. Body size and mass at metamorphosis has been widely demonstrated to influence future fitness in amphibians, improving survival and performance (Szekely et al. 2020). This carry-over effect reduces the potential for predation, and allows individuals to remain competitive and achieve earlier reproductive maturity (Gomez-Mestre and Tejedo 2003; Semlitsch et al. 1988; Wu and Kam 2009).Tadpoles in our salinity treatments were larger at metamorphosis than those raised in rainwater (control), suggesting that if *L. aurea* exposure to salinity induces a negative stress response, then it is likely at salinity levels greater than those tested in our study.

There has been limited investigation into the post-metamorphic locomotor performance of individuals exposed to salinity as tadpoles. We found that rearing tadpoles in water with elevated salinity positively affected jumping ability in *L. aurea* post-metamorphosis in half of the salinity treatments compared to rainwater controls. Locomotor performance is suggested to contribute to survival potential and early growth in the adult stage (Álvarez and Nicieza 2002) and is known to be influenced by environmental conditions during the larval stage (Álvarez and Nicieza 2002; Niehaus et al. 2006). We thus infer a positive impact on lifetime fitness due to exposure to some levels of salinity, another potential hormetic response. Similar examples are found in the Iberian painted frog (*Discoglossus galganoi*), where jumping ability was affected by the temperature and diet that individuals were exposed to as tadpoles (Álvarez and Nicieza 2002). Negative effects of stress induced in tadpoles of *L. aurea* have also been shown to carry over to the post-metamorphic phase, with increased tadpole density shown to reduce immune fitness in post-metamorphic frogs (Clulow et al. 2015). Such effects of various environmental stressors in the tadpole phase on post-metamorphic frogs remains an understudied area and worthy of further investigation, especially through to the reproductive stage of frogs.

## Conclusion

While the use of elevated water salinity has important potential in the fight against chytrid in the landscape of threatened amphibians, understanding the impact of target salt concentrations on the amphibian larval stage is critical. Our study found that previously published target salt concentrations of up to 4.5 ppt for chytrid control (Clulow et al. 2018; Stockwell et al. 2012, 2015) are unlikely to impact the survival and development of tadpoles in our model species, *L. aurea*. We demonstrated a positive association between salinity and body mass up to a concentration of 6 ppt, possibly a hormetic response in this species to low levels of salt. We also found that locomotor performance in post-metamorphic frogs was either no worse or, in some cases, significantly better for tadpoles raised in salinity treatments compared with the rainwater control. This suggests that salinity up to 6 ppt may have a beneficial effect for this species, which represents only a small increase in salinity above freshwater levels (1 ppt) and remains within the concentration range for coastal wetlands (1 ppt–12 ppt) and well below the salinity of seawater (upwards of 35 ppt). Thus, we have shown that salt concentrations known to inhibit chytrid transmission and reduce infection loads in juvenile and adult *L. aurea* frogs (Clulow et al. 2018; Stockwell et al. 2012, 2015) are unlikely to negatively impact the species at the larval phase at least in the coastal population we tested. Further research is of course needed to determine the suitability of such an approach for chytrid-susceptible anurans in freshwater landscapes. However, these findings suggest that adding low doses of salt (up to 6ppt) to artificial aquatic environments maybe a valid approach to mitigating chytrid impacts in the landscapes of *L. aurea* and similar coastal amphibians threatened with the disease, cognisant of the potential impacts at the ecological community scale for other organisms.

## Data Availability

Data is deposited at https://doi.org/10.6073/pasta/be8cf6c12a7b83985588a454421a0795.
